# Sepsis Cards and Facts: A Simple Way to Increase Sepsis Bundle Compliance

**DOI:** 10.7759/cureus.3245

**Published:** 2018-09-04

**Authors:** Leoh Leon, Nicholas Kramer, Latha Ganti, Kendra Amico, Larissa Dub, David Lebowitz, Javier Rosario, Bethany Ballinger

**Affiliations:** 1 Emergency Medicine, University of Central Florida College of Medicine, Orlando, USA

**Keywords:** quality improvement, sepsis

## Abstract

Objective

The objective of this study was to improve sepsis bundle compliance via an educational intervention in our emergency department (ED).

Methods

This was a before and after study. Historical data on sepsis bundle compliance was obtained from our quality officer. Data were collected for 30 consecutive days to compare sepsis bundle compliance rates before and after the intervention. Descriptive statistics were compiled, and the z-test for proportions was used to calculate statistical significance.

The intervention was two-fold: 1) a bright yellow card with sepsis criteria listed was posted on all ED workstation computers and 2) there was a daily email blast for one month with “sepsis facts.” These email blasts were short pearls that highlighted the importance of recognizing and treating sepsis.

Results

The sepsis bundle compliance rates in the month prior to the intervention was 38%. In the month during the targeted intervention, the compliance rate increased to 56%. There was a statistically significant increase in bundle compliance rates during the intervention (p=0.0399).

We also administered a survey to the ED attendings and residents following the completion of the study to assess whether they perceived that our intervention was helping them increase compliance with ordering the sepsis bundle. The response rate was 94%. To the question “Did you feel the sepsis cards placed on the workstations make you more likely to consider sepsis earlier in patients under your care in the emergency department?” 70% answered agree or strongly agree. To the question “Were you more likely to order the sepsis bundle after receiving the daily "Sepsis Facts"?” 29% were neutral while 59% answered agree or strongly agree. Finally, to the question “Did you feel the sepsis cards and "sepsis facts" help you improve the care of Septic patients in the emergency department?” 76% answered agree or strongly agree.

Conclusion

Sepsis criteria reminders and email blasts highlighting the importance of treating and recognizing sepsis can improve compliance with sepsis bundle ordering within the emergency department.

## Introduction

Sepsis is defined as life-threatening organ dysfunction caused by a dysregulated host response to infection. Over 750,000 cases of sepsis occur annually in the United States (US), with the number and rate of hospitalizations tripling over the last two decades and continuing to climb each year [1]. Sepsis accounts for 1.3 million hospital stays per year in the US (3% overall). Early treatment of sepsis has been associated with improved outcomes; for example, the administration of an effective antibiotic within the first hour of documented hypotension in sepsis was associated with a survival rate of 79.9%, and each hour of delay resulted in a 7.6% decrease in survival [2]. Therefore, sepsis screening and early, aggressive care is vital to increasing survival, improving morbidity, and decreasing the overall hospital length of stay [3-5]. Improving sepsis care can also affect health care costs, as sepsis is the most expensive condition treated in US hospitals, at a cost of $23.7 billion in total health care expenditures in 2013 (6.2% of total national costs) [3].

A 2017 study showed that sepsis patients with bundle compliance have a lower mortality rate at 21.3% versus 25.4% in the noncompliant group (CI, 1.3–6.9%) [6]. Despite the evidence that the early recognition and management of sepsis improves patient outcomes, we found that compliance with ordering the sepsis bundle at our institution was low and inconsistent. The objective of our study was to design a quality improvement project to improve sepsis bundle compliance in our emergency department (ED).

## Materials and methods

Study design

We performed quality improvement (QI) before and after the study. De-identified historical data on sepsis bundle compliance were obtained from our quality officer, including the month before the intervention and six months afterward. In our institution, the sepsis bundle consists of: 1) blood cultures before antibiotics, 2) antibiotics within one hour of recognition, 3) serum lactate level at presentation with a three-hour repeat if elevated >2 mmol/L, and 4) crystalloid fluid resuscitation of 30 cc/kg if lactic > 4 mmol/L and/or spontaneous bacterial peritonitis (SBP) < 90 mmHg. We compared sepsis bundle compliance rates before and after the intervention. As no personal health information (PHI) was accessed for this QI project, the project was considered exempt from our Institutional Review Board.

Study setting and population

The study was conducted at a level II trauma center that is home to our medical school’s emergency medicine residency program. The ED sees over 80,000 visits annually, of which over 900 are for sepsis.

Measures

**Figure 1:**
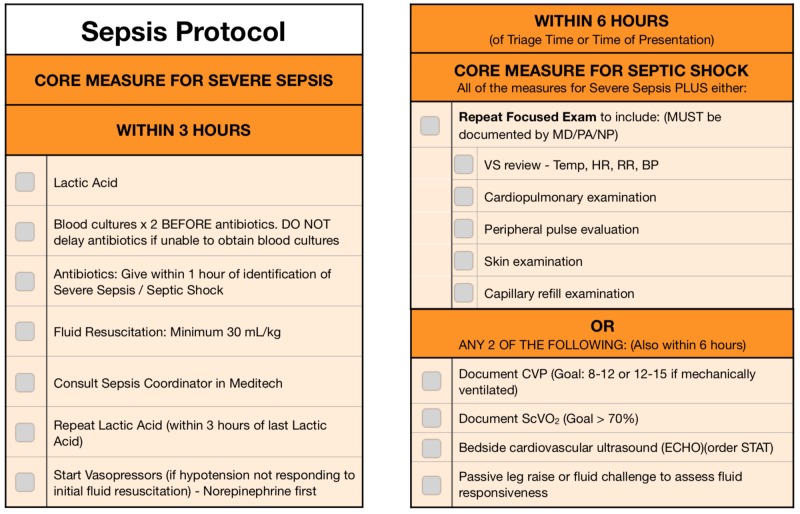
Sepsis Card MD = Doctor of Medicine; PA = Physician Assistant; NP = Nurse Practitioner; HR = Heart Rate; RR = Respiratory Rate; BP= Blood Pressure; CVP = Central Venous Pressure; ScVo2 = Central Venous Oxygen Saturation ECHO= Echocardiography

The intervention was twofold: First, a bright card with sepsis criteria listed was posted on all ED workstation computers (Figure 1) for 30 consecutive days. This was followed by a daily email blast sent to all ED providers for one month with “Sepsis Facts” (Table 1). These email blasts were engaging pearls that highlighted the importance of recognizing and treating sepsis.

**Table 1:** Sepsis Facts ACEP = American College of Emergency Physicians; MRSA = Methicillin-Resistant Staphylococcus Aureus; CMS = Centers for Medicare & Medicaid Services; COPD = Chronic Obstructive Pulmonary Disease

SEPSIS FACTS
26%-48% of severe sepsis patients are readmitted within 180 days [7]
12.2% of sepsis patients are readmitted within 30 days, making sepsis the leading cause of readmission [8]
ACEP defines severe sepsis as lactate >2 or organ dysfunction [9]. Remember - if a patient has a lactate >2 and it's not sepsis, be sure to document why not
ACEP defines septic shock as severe sepsis with hypoperfusion despite adequate fluid resuscitation or a lactate >4 [9]
The average cost per hospital stay for sepsis is $18,400 [3]
Sepsis accounts for 1.3 million hospital stays per year in the US (3% overall) [3]
Sepsis bundle elements have greater impact when performed simultaneously as compared to in isolation [10]
Sepsis is the largest killer of children and newborn infants in the world [10]
As many as 80% of sepsis deaths could be prevented with rapid diagnosis and treatment [5]
In 2009, the in-hospital mortality rate for septicemia was about 16 percent—more than 8 times higher than other stays (2.0 percent). This was unchanged from 2000 [11]
From 1993 to 2009, septicemia-related hospital stays more than doubled, increasing by 153 percent overall, for an average annual increase of 6 percent [11]
E. Coli is the most common specifically-identified organism for patients with a principal diagnosis of septicemia. MRSA is the most common for patients with a secondary diagnosis of septicemia. However, more than half of septicemia cases have no organism identified on the discharge record [11]
Sepsis accounts for $14.5 billion in Medicare costs, making it the number one most expensive cost billed to Medicare (5 billion more than the next most expensive condition) [3]
Medicare is the predominant payer for sepsis covering 61.2% of the costs [3]
The sepsis core measure clock typically starts at time of triage [9]
Each hour of delay in antimicrobial administration over the ensuing 6 hours after sepsis identification is associated with an average decrease in survival of 7.6% [5]
Administration of an effective antibiotic within the first hour of documented hypotension in sepsis was associated with a survival rate of 79.9% [5]
To be compliant with Sepsis CMS core measures, you must have ALL of the following in the first 3 hours after presentation: 1) serum lactate, 2) blood cultures prior to antibiotics, 3) broad-spectrum antibiotic therapy, and 4) 30 ml/kg crystalloid for hypotension or lactate >4 [9]
Sepsis is the number one most expensive condition treated in US hospitals, 2013, costing $23.7 billion in health care expenditures in 2013 (6.2% of total national costs) [3]
80% of patients diagnosed with sepsis developed the condition outside of the hospital [12]. CLINICAL PRACTICE IDEA - Order blood cultures (and consider ordering the sepsis bundle) on ALL COPD and respiratory failure patients since they will likely receive antibiotics and may decompensate inpatient
6% of all deaths have sepsis listed among the causes of death [12]
The mean total direct cost for patients with severe sepsis or septic shock is significantly lower in septic patients that are bundle compliant compared to those that weren't ($14,845 versus $20,056 [6]
Sepsis patients with bundle compliance have a lower mortality rate at 21.3% versus 25.4% in the noncompliant group (CI, 1.3–6.9%) [6]

Data analysis

Descriptive statistics were compiled, and the z-test for proportions was used to calculate statistical significance.

## Results

The sepsis bundle compliance rate in the month prior to the intervention was 38%. In the month during the targeted intervention, the compliance rate increased to 56%. There was a statistically significant increase in bundle compliance rates during the intervention (p=0.0399). The intervention demonstrated continued improvement, with the bundle compliance rate at six months post-intervention being 79%.

**Figure 2:**
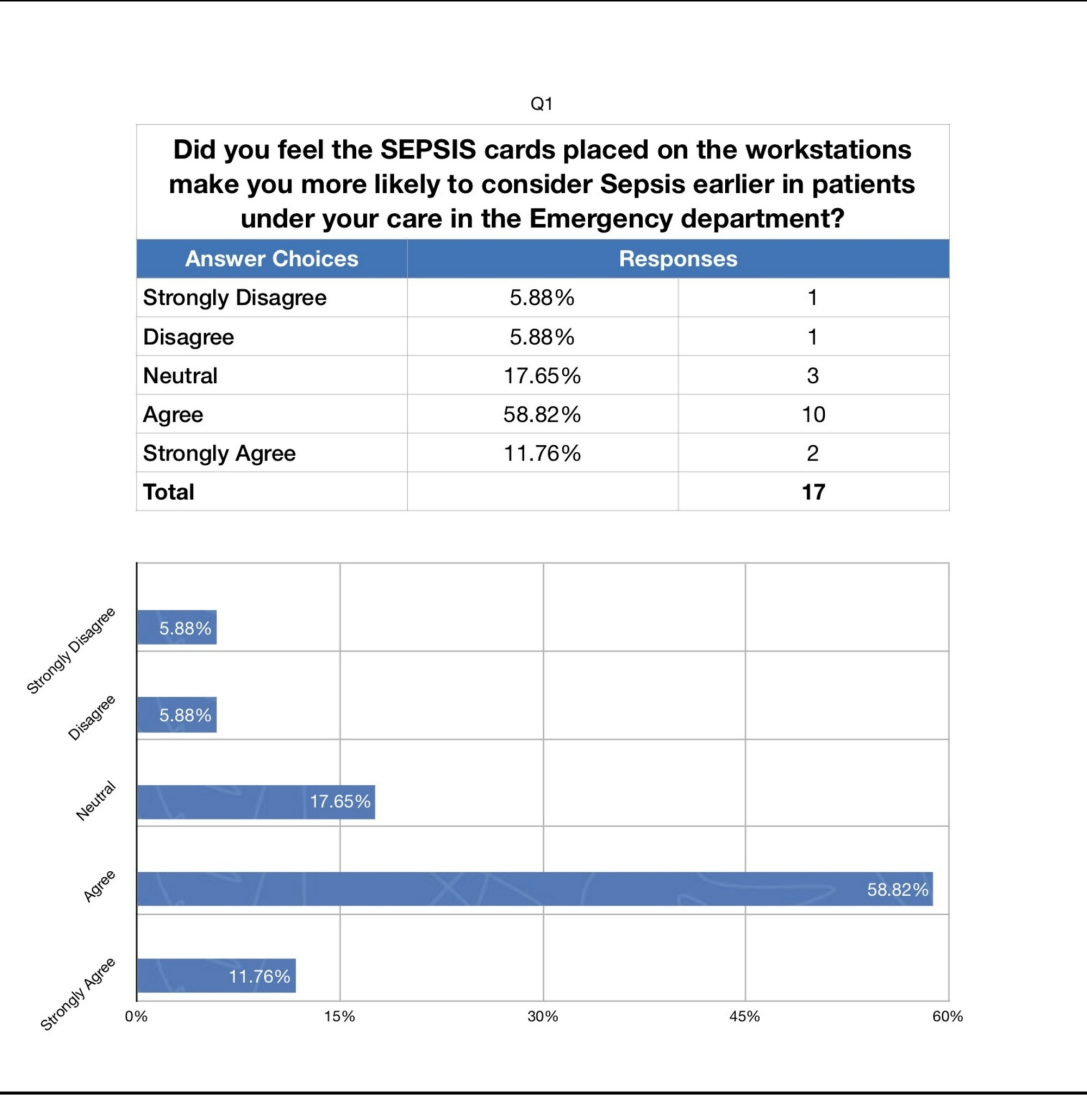
Survey Results: Sepsis Cards

**Figure 3:**
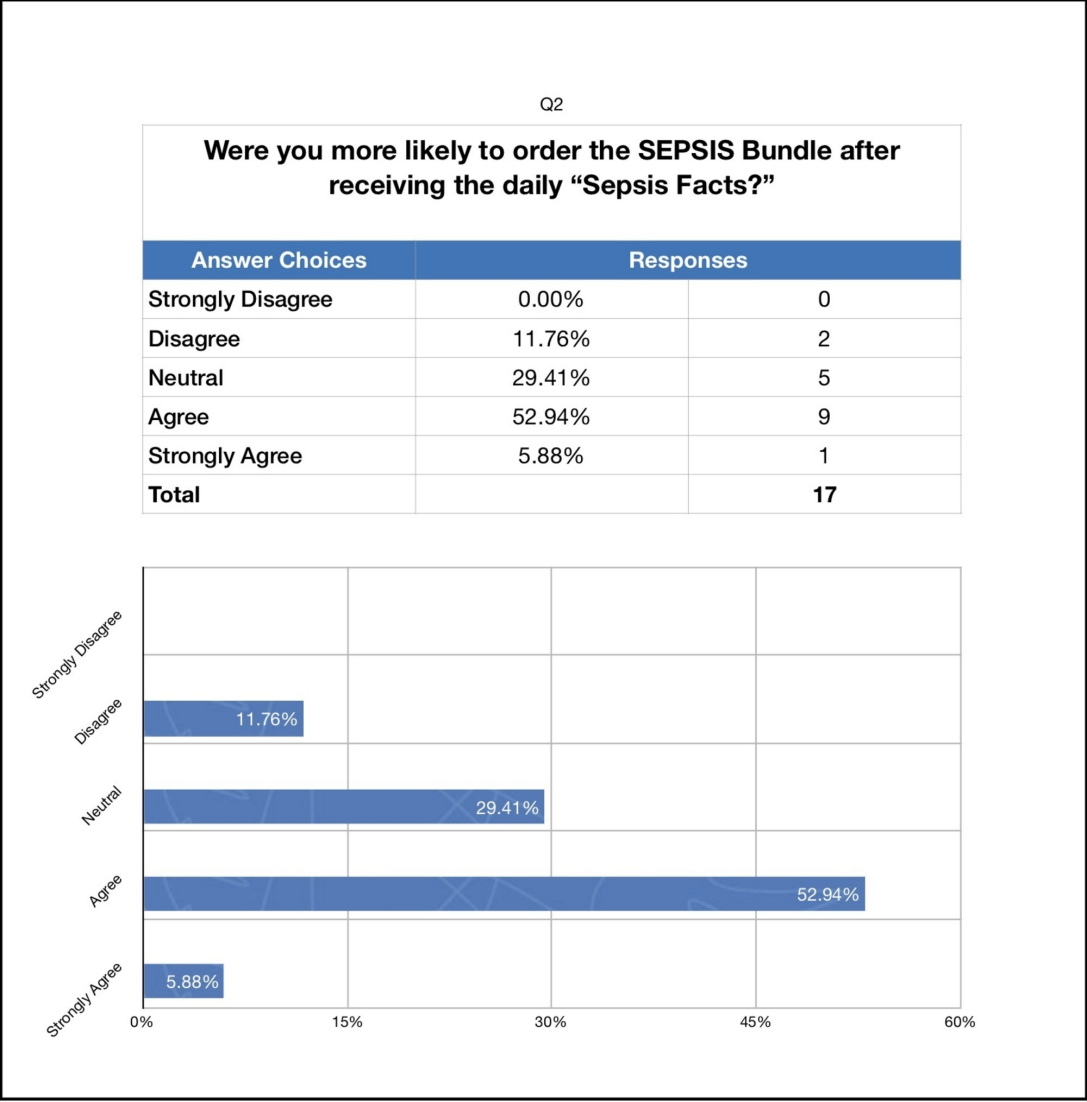
Survey Results: Fun Facts

**Figure 4:**
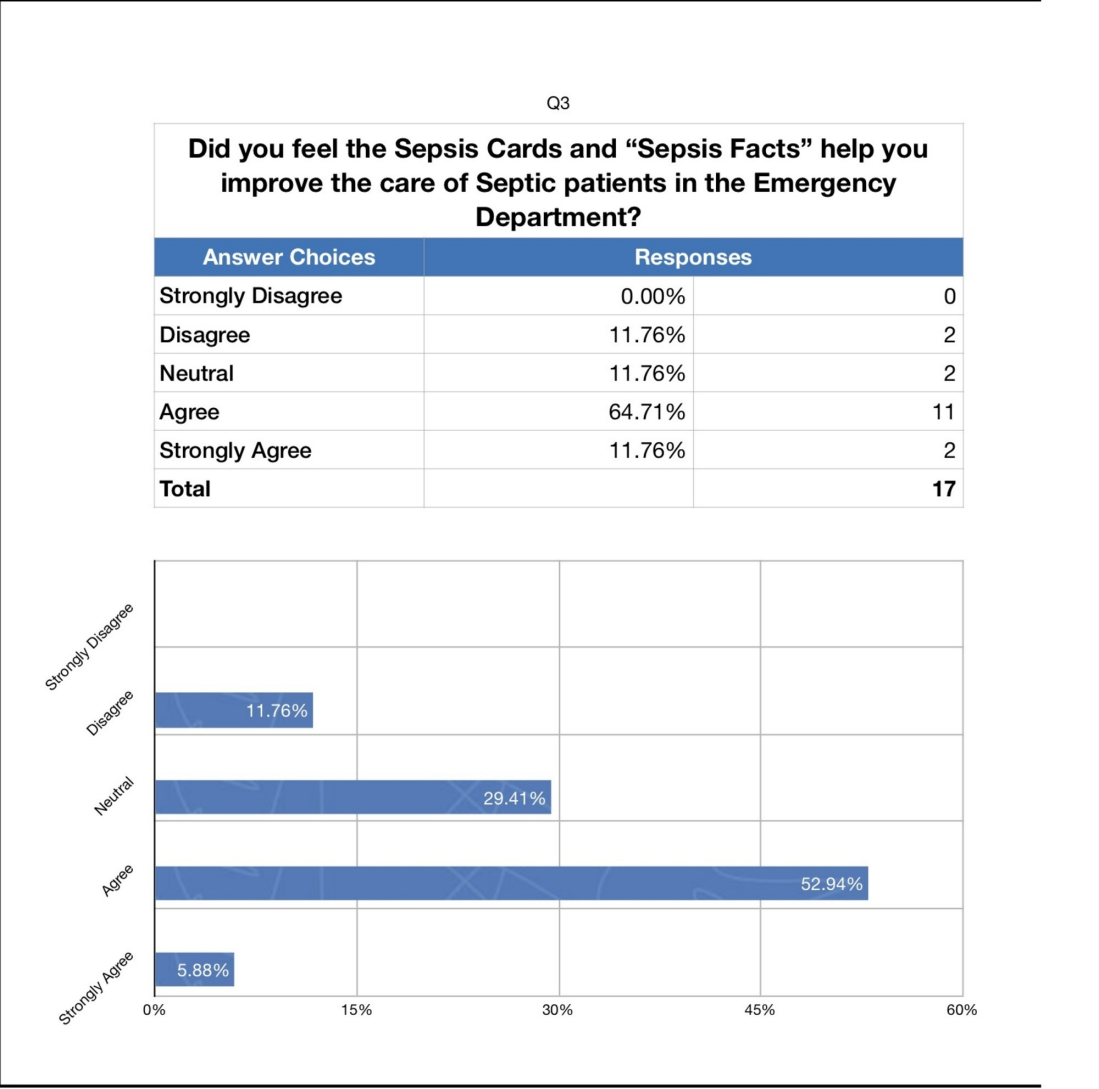
Survey Results: Improved Care

We also administered a survey to the ED attendings and residents following the completion of the study to assess whether they perceived our intervention as helpful in increasing their compliance with ordering the sepsis bundle. The response rate was 94% (Figures 2-4).

To the question “Did you feel the sepsis cards placed on the workstations make you more likely to consider sepsis earlier in patients under your care in the emergency department?” 70% answered agree or strongly agree. To the question “Were you more likely to order the sepsis bundle after receiving the daily "sepsis facts’?” 29% were neutral while 59% answered agree or strongly agree. Finally, to the question “Did you feel the sepsis cards and ‘sepsis facts’ help you improve the care of septic patients in the emergency department?” 76% answered agree or strongly agree.

## Discussion

In this before and after study, we demonstrated that small interventions can have meaningful results. There is significant literature involving the importance of recognizing sepsis; however, we were unable to find any studies that created a specific intervention to increase sepsis awareness for the early implementation of the sepsis bundle.

Studies that incorporate screening methods to identify specific patients in the ED have previously been shown to produce significant results. For example, a suicide screening and detection for all patients in the ED identified nearly twice as many patients at risk for suicide [13]. Additionally, a systematic review that looked at the effectiveness of interventions aimed at increasing handwashing in healthcare workers found that interventions based on an assessment of individual and organizational barriers to behavior change were found to be more effective, and multifaceted interventions were generally more effective than single interventions [14].

Our daily emails and bright yellow cards proved to have a significant effect on the awareness and identification of septic patients, leading to the early implementation of the sepsis bundle. We recognize that any “reminder” will result in behavior change, at least temporarily. For example, daily reminders have proved to be very effective for medication compliance [15]. Similarly, our daily reminders led to an increased identification of patients who met the sepsis criteria. Seventeen ED physicians were surveyed regarding the impact of intervention: the majority agreed that they were more likely to consider sepsis earlier, improve their care of septic patients, and felt that the daily “sepsis facts” led them to utilize the sepsis bundle more often.

Our intervention led to an 18% increase in bundle compliance in just one month [16]. Our small intervention has led to an increased awareness and interest in sepsis and has slowly become part of the “culture” in our emergency department. We have noticed that not only physicians but also the nursing staff has also developed an “eye” for the identification of septic patients and are eager to alert physicians in order to begin the appropriate treatment, which, in turn, saves lives.

Finally, from an administrative standpoint, early identification and bundle compliance has been shown to decrease the length of hospitalization, rates of readmission, and overall mortality. Mortality was lower at hospitals that had high compliance with the resuscitation bundle (29.0% vs. 38.6%) and, less dramatically, at those that had high compliance with the management bundle (32.3% vs. 33.8%). For every 10% increase in compliance with the resuscitation bundle, length of stay in both the hospital and intensive care unit decreased by 4% [17].

## Conclusions

In this before-and-after study, we demonstrated that small interventions can have meaningful results. There is significant literature involving the importance of recognizing sepsis; however, we were unable to find any studies that created a specific intervention to increase sepsis awareness for the early implementation of the sepsis bundle.
